# Pathology of Cholera

**Published:** 1867-05

**Authors:** W. Duff Greene

**Affiliations:** Mount Vernon, Ill.


MOUNT VERNON, Ill., *March 26, 1867.*

Prof. N. S. DAVIS:—

*Dear Sir*,—The probable return of cholera to this country during the present year makes it necessary that physicians should devote some time and thought to devising a better mode of treatment than has been adopted heretofore. This better mode of treatment must be based upon changed or rather settled views of the pathology of cholera.

Without troubling you with a lengthy exposition of my views, let me tell you in a few words what they are. I have held them for a number of years, communicating them freely to my neighboring physicians, but have had few opportunities of putting them to the test. They are these: The fatality of cholera is due to the dessication of mucous lining of the bronchial tubes and pulmonary vessicles, whereby the proper oxygenation and decarbonization of the blood are prevented. That these conditions actually exist would not be controverted by any one, but the why has not yet been fully ascertained. Dr. Johnson comes more nearly to an explanation, when he attributes it to paralysis of the capillaries of the lungs. Does not the theory of *dessication* more fully account for it? Fluids do not readily permeate dried membranes—in cholera this condition actually exists—that is, the blood of a cholera patent, from some cause, does not receive the full benefit of due and proper oxygenation. Now, if it were fully ascertained by *post mortem* examinations that the assumed condition of the pulmonary mucus linings does actually exist, would it not be admitted that the hindrance to the process of *osmosis* would be sufficient to account for the phenomena of cholera? Farther, although the investigations of pathologists have not positively shown this condition to exist, (perhaps from their attention not having been directed to it) do not some of the ascertained facts give considerable plausability to this theory? The upper air-passages are always dried, as evidenced by the dry hissing voice and by the actual condition of the nostril and mouth, as seen in life; and may we not assume that the same condition extends to the pulmonary vesicles. Is not this condition of the same parts still further proven, or at least rendered probable, by the fact that, while there is no mechanical hindrance to the free entrance of air into, and its diffusion over, the interior surface of the pulmonary linings, it comes from the lung almost unchanged by expiration, having lost but little oxygen and gained but little carbonic acid gas. See Davy & Bamel. What so readily accounts for this unchanged condition of inspired air, as that the pulmonary linings are dessicated (by the unknown cause of cholera) to such an extent that the function of *osmosis* cannot be fully and properly performed.

Now, theories are of but little avail if they do not point out a mode of treatment. But does not this theory point clearly to a rational mode of treatment? It seems to me that the inhalation of moist vapor, medicated as each practitioner might prefer, would be a necessary and rational mode of treatment. Would not, again, the exclusion of larger doses of opium from our prescriptions follow as a necessary consequence, for, if I am not mistaken, it is an ascertained fact that the pulmonary linings are found in a dried condition in cases of poisoning by that article.

Would not this theory suggest the free use of such remedies at possess the property of oxygenating the blood when introduced therein through the stomach? Concentric friction of the extremities, as part of "Marshall Hall's Ready Method," would certainly suggest itself to the mind of every thinking physician as an important adjuvant. Would not, again, inhalation of vapor charged with iodine and other agents known to be destructive to vegetable life, immediately suggest itself to those gentlemen who have adopted the "Fungus Theory" of cholera?

These random thoughts I have hurriedly thrown together, and hope that you will give them some thought and consideration, and should you think proper, submit them to your Medical Society at its next meeting; and although they may be productive of no immediate good, they will certainly afford food for thought, and possibly be productive of ultimate good results.

I need offer you no apology for thus intruding these thoughts upon you without a personal acquaintance with you, for your known interest in, and devotion to, science is a sufficient warrant to me for so doing.

Respectfully, etc.,

W. DUFF GREEN, M.D.

